# Artificial Intelligence-Aided Tooth Detection and Segmentation on Pediatric Panoramic Radiographs in Mixed Dentition Using a Transfer Learning Approach

**DOI:** 10.3390/diagnostics15202615

**Published:** 2025-10-16

**Authors:** Serena Incerti Parenti, Giorgio Tsiotas, Alessandro Maglioni, Giulia Lamberti, Andrea Fiordelli, Davide Rossi, Luciano Bononi, Giulio Alessandri-Bonetti

**Affiliations:** 1 Unit of Orthodontics and Sleep Dentistry, Department of Biomedical and Neuromotor Sciences (DIBINEM), University of Bologna, Via San Vitale 59, 40125 Bologna, Italy; alessandro.maglioni@unibo.it (A.M.); giulia.lamberti2@studio.unibo.it (G.L.); andrea.fiordelli2@unibo.it (A.F.); giulio.alessandri@unibo.it (G.A.-B.); 2 Department of Computer Science and Engineering (DISI), University of Bologna, Mura Anteo Zamboni 7, 40126 Bologna, Italy; giorgio.tsiotas@unibo.it (G.T.); daviderossi@unibo.it (D.R.); luciano.bononi@unibo.it (L.B.)

**Keywords:** artificial intelligence, deep learning, panoramic radiography, mixed dentition, image interpretation, pediatric dentistry

## Abstract

**Background/Objectives**: Accurate identification of deciduous and permanent teeth on panoramic radiographs (PRs) during mixed dentition is fundamental for early detection of eruption disturbances, yet relies heavily on clinician experience due to developmental variability. This study aimed to develop a deep learning model for automated tooth detection and segmentation in pediatric PRs during mixed dentition. **Methods**: A retrospective dataset of 250 panoramic radiographs from patients aged 6–13 years was analyzed. A customized YOLOv11-based model was developed using a novel hybrid pre-annotation strategy leveraging transfer learning from 650 publicly available adult radiographs, followed by expert manual refinement. Performance evaluation utilized mean average precision (mAP), F1-score, precision, and recall metrics. **Results**: The model demonstrated robust performance with mAP_0.5_ = 0.963 [95%CI: 0.944–0.983] and macro-averaged F1-score = 0.953 [95%CI: 0.922–0.965] for detection. Segmentation achieved mAP_0.5_ = 0.890 [95%CI: 0.857–0.923]. Stratified analysis revealed excellent performance for permanent teeth (F1 = 0.977) and clinically acceptable accuracy for deciduous teeth (F1 = 0.884). **Conclusions**: The automated system achieved near-expert accuracy in detecting and segmenting teeth during mixed dentition using an innovative transfer learning approach. This framework establishes reliable infrastructure for AI-assisted diagnostic applications targeting eruption or developmental anomalies, potentially facilitating earlier detection while reducing clinician-dependent variability in mixed dentition evaluation.

## 1. Introduction

Artificial intelligence (AI) has emerged as one of the most rapidly evolving innovations in healthcare, with increasingly relevant applications in dentistry. Advances in machine learning algorithms, deep learning (DL) architectures, and large language models (LLMs) have enabled AI to support multiple aspects of dental care, from diagnosis and treatment planning to patient communication. Recent studies have demonstrated the utility of AI across multiple domains, ranging from caries detection, periodontal disease assessment, pediatric dentistry, and endodontic procedures, exhibiting encouraging accuracy and efficiency, despite considerable variation in model performance across different studies [1].

The integration of AI in radiographic interpretation represents one of the most promising applications in dentistry, particularly for enhancing image interpretation speed and diagnostic accuracy. Moreover, looking toward future perspectives, AI technologies hold considerable potential for transforming patient communication and engagement in clinical decision-making processes. The widespread adoption of LLM-based chatbots has reshaped healthcare information dissemination, with tools such as ChatGPT demonstrating promising performance across various dental fields [2,3,4,5,6], and potentially challenging traditional search engines like Google as primary resources for patient healthcare inquiries. However, a recent systematic review emphasizes the critical need for further research to assess LLM content reliability and its clinical impact, given the inherent risk of generating inaccurate information [7].

The monitoring of the transition from the mixed to the permanent dentition is an essential component of pediatric dental care and presents unique challenges that are particularly well-suited for AI-assisted solutions. Tooth eruption is a dynamic and sequential developmental process with the transition from primary to permanent dentition occurring during the mixed dentition period, generally between 6 and 12 years of age. This period is characterized by the interplay of primary tooth exfoliation and permanent tooth eruption. Accurate tooth numbering systems, such as the Federation Dentaire Internationale (FDI), are essential for proper diagnosis, communication, and treatment planning.

Early identification of tooth developmental and eruption anomalies during the mixed dentition period is crucial for optimal patient care. Conditions including agenesis, supernumerary teeth, delayed or ectopic eruption can significantly disrupt the normal sequence of dental development, potentially leading to space discrepancies, malocclusions or tooth impactions. Timely detection enables the proactive management of developmental anomalies and the implementation of interceptive strategies when needed, such as orthodontic space management, or strategic extractions capable of redirecting aberrant eruption patterns toward physiological pathways and potentially reducing the likelihood of complex interventions later in development [8,9].

Panoramic radiographs (PRs) represent a widely used diagnostic tool for mixed dentition assessment, providing comprehensive visualization of dental development and identification of eruption disturbances in a single exposure [9]. Beyond its routine diagnostic applications, PRs play a fundamental role in dental age estimation, offering reliable information on tooth development chronology and eruption patterns that prove critical in pediatric dentistry, orthodontics, and forensic contexts. Recent evidence, including a 2025 systematic review and meta-analysis, has further demonstrated the potential of DL to enhance age estimation from PRs, highlighting the promise of AI-assisted approaches in this domain [10]. The ability of PR to simultaneously evaluate the position, morphology, and teeth developmental stage, combined with emerging DL applications, underscores its importance as both a first-line diagnostic tool and a valuable adjunct for guiding clinical decision-making and interceptive interventions during the mixed dentition period.

However, accurate interpretation of mixed dentition PRs remains highly dependent on clinician experience, particularly given the variable coexistence of deciduous and permanent teeth at different developmental stages and the frequent overlap of anatomical structures [11]. Implementing a fully automated approach could overcome these limitations while simultaneously enhancing both diagnostic accuracy and clinical efficiency.

While recent advances in DL have yielded promising results for automated tooth detection and numbering in PRs of permanent dentition [12,13,14], research focusing specifically on mixed dentition remains limited, and several critical gaps persist in the current literature [15,16,17,18,19]. Among the studies addressing mixed dentition, most have predominantly employed bounding-box detection approaches [15,16,17,18], with limited exploration of detailed polygonal segmentation [19]. Furthermore, none have reported performance stratification by dentition type or provided in-depth error characterization. Additionally, current investigations have not adequately explored the potential of state-of-the-art YOLO architectures, particularly YOLOv11 [20], nor have they evaluated innovative methodologies to optimize pediatric annotation workflows, thereby failing to address the inherent challenges associated with tooth recognition in mixed dentition scenarios.

This study addresses these limitations by introducing a novel YOLOv11-based framework that integrates simultaneous detection and segmentation capabilities specifically optimized for PRs in mixed dentition. The key methodological innovation lies in implementing a hybrid pre-annotation strategy that leverages transfer learning from a publicly available adult radiographic dataset to automatically generate provisional tooth labels for pediatric images, followed by systematic expert manual refinement. Our framework incorporates comprehensive performance assessment, including stratified analyses for deciduous and permanent dentition as well as detailed error characterization. This integrated methodology represents a substantial step forward in the development of high-precision AI systems specifically tailored for pediatric dental imaging applications.

The null hypothesis was that our novel YOLOv11-based approach could not achieve acceptable performance in automated tooth detection and segmentation in mixed dentition PRs. By addressing the current gaps in pediatric dental AI research, this study aims to provide a robust foundation for the clinical implementation of AI-assisted mixed dentition analysis, ultimately contributing to more accurate, efficient, and standardized pediatric dental care. This work contributes to the field in three relevant ways:

- task integration: a YOLOv11-based framework that jointly performs detection, enumeration, and polygonal instance segmentation in a single pass, tailored to mixed dentition;
- efficient pediatric labeling: a hybrid pre-annotation strategy that reduces manual burden while preserving accuracy;
- dentition-aware reporting: comprehensive stratification of performance by dentition type and error profiling, highlighting clinically relevant failures that prior studies did not examine.

## 2. Materials and Methods

### 2.1. Study Design

This retrospective study was conducted at the Unit of Orthodontics and Sleep Dentistry, Department of Biomedical and Neuromotor Sciences (DIBINEM), in collaboration with the Department of Computer Science and Engineering (DISI), University of Bologna, Italy. The study was conducted in accordance with the Declaration of Helsinki and approved by the Institutional Ethics Committee CE-AVEC (approval number: 293-2024-AUSLBO; 16 October 2024). Informed consent was obtained from all subjects involved in the study. Personal data were processed in accordance with the applicable data protection legislation (in particular Regulation (EU) 2016/679 and Legislative Decree No. 196 of 30 June 2003, as subsequently amended and supplemented). Study conduct and reporting adhered to the Checklist for Artificial Intelligence in Medical Imaging guidelines [21] and to the STROBE (Strengthening the Reporting of Observational Studies in Epidemiology) guidelines [22] to ensure transparency, reproducibility, and methodological rigor (Table S1).

### 2.2. Dataset Description

A total of 250 PRs were retrospectively selected from patients who underwent routine diagnostic imaging between 2017 and 2024 at the School of Dentistry, DIBINEM, University of Bologna, Italy. Eligible patients were aged 6–13 years presenting mixed dentition. The age range was specifically chosen to encompass the critical transitional period when both deciduous and permanent teeth coexist, allowing comprehensive evaluation of diverse developmental stages and tooth morphologies essential for robust model training. Exclusion criteria comprised: (1) poor image quality with blurred or distorted tooth contours that could compromise accurate annotation (image quality was defined as clear visualization of tooth contours, absence of motion artifacts, and sufficient contrast to discriminate teeth from surrounding bone); (2) presence of supernumerary teeth that might confound automated tooth identification algorithms; (3) cystic lesions or pathological conditions affecting normal dental anatomy; and (4) active fixed orthodontic appliances, which could introduce metallic artifacts and obscure tooth boundaries.

The sample size was determined based on data availability within the institutional archive and was deemed sufficient to allow robust model training and evaluation.

All images were acquired using a Planmeca ProMax panoramic system (Planmeca Oy, Helsinki, Finland) with standardized exposure parameters (60–64 kVp, 5 mA, 12 ms) and patient positioning using guide lights and chin stabilization. Images were exported in PNG format at 2454 × 1304 pixel resolution, providing optimal balance between image quality and computational efficiency for DL applications.

### 2.3. Labeling Protocol

**Figure 1:**
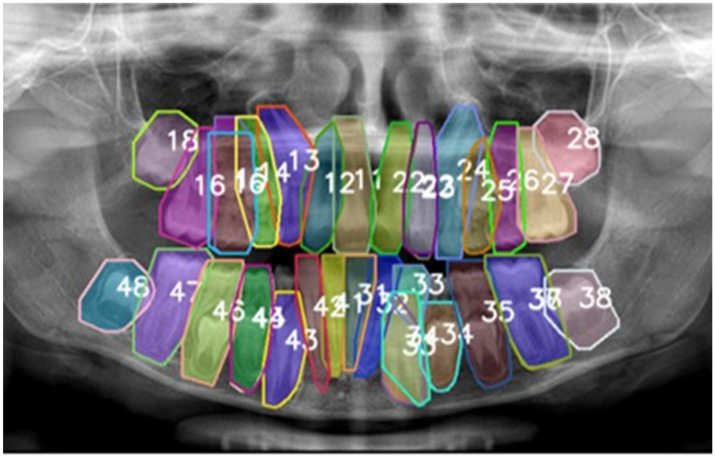
The pre-annotation phase was conducted using the preliminary model trained on the adult dataset DENTEX in order to streamline the subsequent labeling process. The numbers represent the type of tooth according to the FDI numbering system. Each tooth was labeled with a unique, non-semantic color.

As a preliminary step, 650 annotated PRs from adult patients were obtained from the publicly available Dental Enumeration and Diagnosis on Panoramic-X-rays (DENTEX) dataset, released as part of the International Conference on Medical Image Computing and Computer-Assisted Intervention (MICCAI) 2023 [23,24]. This adult dataset served as the training foundation for developing a preliminary DL model specifically designed for permanent tooth identification and enumeration. The resulting pre-trained model was subsequently applied to automatically pre-annotate the 250 pediatric PRs, thereby establishing initial tooth boundaries and classifications to streamline the subsequent manual labeling task (Figure 1).

**Figure 2:**
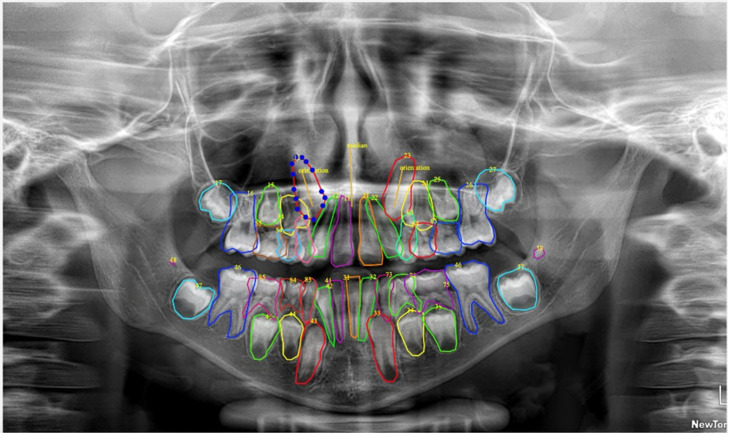
Labeling of the pediatric dataset was performed using a customized open-source tool. Annotations included deciduous and permanent teeth and were made using a segmented line to outline the shape of the tooth The numbers represent the type of tooth according to the FDI numbering system. Each tooth was labeled with a unique, non-semantic color.

Given the inherent limitations of adult-trained models in recognizing deciduous teeth and developing permanent teeth at various stages of formation, a comprehensive manual annotation phase was essential. A customized open-source labeling tool was employed to facilitate expert-driven manual annotation according to the FDI tooth numbering system. This phase specifically focused on accurately identifying and classifying deciduous teeth and developing permanent teeth that were either misclassified or unrecognized by the adult pre-trained model (Figure 2).

All manual annotations were performed by a specialist in orthodontics with more than fifteen years of clinical experience. The specialist systematically reviewed each pre-annotated image, correcting automated classifications where necessary and manually annotating previously undetected dental elements. All labeled images underwent independent verification by a senior orthodontist with more than thirty years of clinical experience. Any discrepancies between the two evaluators were discussed and resolved through consensus. Full agreement was achieved across all tooth classifications and segmentations.

### 2.4. AI Model Architecture and Training

In this study, we employed YOLOv11 (“You Only Look Once”—version 11), a state-of-the-art DL architecture widely adopted for object detection and image segmentation [20]. YOLO is specifically designed to concurrently perform three tasks on a single image:

- detect the location of each object (in our case, each tooth),
- classify the object (e.g., tooth 11, tooth 12, tooth 35, etc.), and
- segment its precise contour (tooth boundary).

The model follows a YOLO-style single-stage design, consisting of three main components: a backbone for feature extraction, a neck (FPN/PAN-like) for multi-scale feature aggregation, and dedicated heads for detection and segmentation.

YOLOv11 is structured as follows:

- backbone: extracts visual features from the input image (e.g., shapes, contours), leveraging convolutional blocks with batch normalization and SiLU activations;
- neck: employs upsampling and concatenation operations to generate multi-resolution feature maps, enabling the combination and enhancement of features across different scales;
- heads:
  ○ detection head: outputs the position, class label, and confidence score for each object. For each grid cell, the detection head predicts bounding-box parameters (tx, ty, tw, th), an objectness score, and class logits. The detection loss combines three components: a bounding-box regression loss (IoU-based), an objectness loss (BCE), and a classification loss (Cross-Entropy or BCE, depending on the parametrization);
  ○ segmentation head: produces pixel-level masks for each detected object using an instance-aware single-shot approach. The head generates a small set of prototype masks and, for each detection, a coefficient vector. The final mask for detection i is obtained by linearly combining the prototypes with the coefficients, followed by a sigmoid activation and cropping according to the bounding box.

This design enables the generation of instance-level segmentation outputs (i.e., one mask per detected tooth), even in cases where teeth are spatially adjacent or overlapping.

### 2.5. Output Layer Configuration

In this study, the model was trained to detect and classify up to 52 tooth types (32 permanent and 20 deciduous teeth, corresponding to mixed dentition).

For each detected tooth, the model provides:

- bounding box coordinates (x, y, width, height),
- class label (e.g., 11, 12, …), and
- an instance-specific segmentation mask.

Unlike architectures that produce separate segmentation maps, the final layer outputs one mask per detected tooth instance, directly associated with the predicted class.

The model architecture was derived from the pre-trained Ultralytics YOLOv11-seg-small backbone, which was subsequently fine-tuned on our mixed-dentition dataset to adapt the network for the specific task of accurate detection and segmentation of both deciduous and permanent teeth.

The dataset was randomly partitioned into training (80%), validation (10%), and test (10%) subsets. The training process utilized 225 PRs, comprising the training and validation portions of our pediatric dataset.

To enlarge the effective training dataset size and improve model generalizability, we incorporated the 650 annotated adult panoramic radiographs from the DENTEX dataset during the initial training phase. This strategic combination of adult and pediatric images substantially increased the anatomical variation encountered during optimization, empirically trying to improve detection performance for late-erupting permanent teeth that may exhibit adult-like morphological characteristics.

The model architecture was modified to include a custom loss function specifically designed to penalize the erroneous identification of multiple classes for the same anatomical tooth position during training. Data augmentation hyperparameters were carefully optimized for dental radiographic analysis: scale transformation (scale) was limited to 0.1 to preserve anatomical proportions, pixel erasure (erase) was set to 0.1 to simulate minor image artifacts, image rotation (degrees) was set to 0.0, while mosaic augmentation was disabled (mosaic = 0.0) to maintain spatial relationships between adjacent teeth. Horizontal flip augmentation was specifically disabled (fliplr = 0.0) to prevent left-right tooth misclassification, which is critical for accurate dental enumeration according to the FDI system.

The training protocol employed a two-stage approach. Initial training was performed on a single NVIDIA RTX 2080 GPU using an intersection over union (IoU) threshold of 0.7 and an initial learning rate (lr) of 0.01. This was followed by a specialized fine-tuning phase exclusively on the pediatric PRs (20 epochs, lr = 0.0005, freeze = 10), enabling more precise deciduous teeth segmentation boundaries while preserving adult tooth morphology capability.

**Figure 3:**
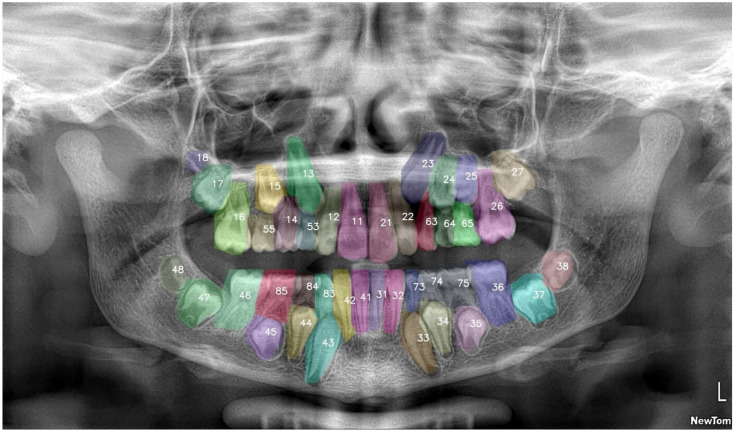
Example of DL model’s performance. The final model was able to recognize deciduous teeth and developing permanent teeth. The numbers represent the type of tooth according to the FDI numbering system. Each tooth was labeled with a unique, non-semantic color.

During inference, the IoU threshold was reduced to 0.1 to minimize prediction duplicates while maintaining detection sensitivity. Training was automatically terminated at epoch 54 through early stopping when peak validation performance (mean average precision, mAP@0.5 = 0.949) was achieved. The model checkpoint demonstrating the highest validation mAP was selected and exported for final evaluation on the independent test set (10% of total dataset) (Figure 3).

### 2.6. Evaluation Metrics & Statistical Analysis

Model performance was evaluated using standard metrics: precision, recall, F1-score, and mAP, offering a comprehensive assessment of detection and localization accuracy. Performance metrics were initially computed on a per-class basis for each individual tooth type, and, subsequently, macro-averaged values were calculated using unweighted averaging across all tooth classes to provide overall performance indicators without bias toward more frequently occurring tooth types. Additional subset analyses were performed by computing separate macro-averages exclusively for deciduous teeth and permanent teeth categories, facilitating targeted evaluation of model performance on each dentition type within the mixed dentition context.

Error pattern analysis was conducted through the construction of a multiclass confusion matrix normalized to percentages, enabling visualization of systematic misclassification patterns and identification of challenging tooth discrimination scenarios.

In addition to the 80/10/10 dataset split, we performed a 5-fold cross-validation to evaluate model robustness and generalization. In each fold, the model was trained from scratch with newly defined training/validation partitions, while the independent pediatric test set remained untouched. Cross-validation results confirmed the stability of the YOLOv11 framework, showing consistently high precision, recall, and mAP values with low variance across folds.

Statistical analyses were performed using the software Python for Windows (Python Software Foundation. Python Language Reference, version 3.1. Available at http://www.python.org, accessed on 5 June 2025).

## 3. Results

The test subset comprised 25 PRs from pediatric patients (mean age 8.4 ± 1.8 years; 14 females, 11 males) and yielded 921 annotated tooth instances, consisting of 810 permanent teeth and 111 deciduous teeth. Tooth classes #71 and #81 were excluded from the analysis due to their extremely low frequency in the dataset, which would have generated unreliable per-class estimates and potentially distorted macro-averaged performance metrics.

For the bounding box detection task, the YOLOv11 model demonstrated robust performance across all tooth categories (Table 1), achieving an overall mAP_0.5_ of 0.963 [95% CI: 0.944–0.983] and a macro-averaged F1-score of 0.953 [95% CI: 0.922–0.965]. This performance was driven by consistently high precision of 0.946 [95% CI: 0.922–0.969] and recall of 0.945 [95% CI: 0.921–0.968], indicating balanced detection accuracy with minimal false positive and false negative predictions.

As can be seen in Table 2, for the segmentation task, the model maintained clinically acceptable performance with mAP_0.5_ = 0.890 [95% CI: 0.857–0.923], precision = 0.893 [95% CI: 0.864–0.922], recall = 0.894 [95% CI: 0.862–0.926], and F1-score = 0.891 [95% CI: 0.862–0.921]. The slightly reduced performance in segmentation compared to detection reflects the increased complexity of precise boundary delineation for irregularly shaped dental structures.

Stratified analysis by dentition type revealed differential model performance between tooth categories. Permanent teeth demonstrated excellent recognition accuracy with an average F1-score of 0.977 [95% CI: 0.971–0.982], while deciduous teeth showed lower but clinically acceptable performance with an average F1-score of 0.884 [95% CI: 0.833–0.934]. This performance discrepancy can be attributed to the morphological variability of deciduous teeth, their smaller size, and the frequent anatomical overlap with adjacent developing permanent teeth.

The three least accurate classifications were identified as the maxillary right deciduous central incisor (#51, F1 = 0.654), the maxillary right deciduous lateral incisor (#52, F1 = 0.704), and the mandibular right deciduous canine (#82, F1 = 0.759). Detailed examination of misclassified cases revealed that these errors predominantly occurred in instances where deciduous teeth exhibited significant overlap with neighboring deciduous elements or adjacent unerupted permanent teeth, thereby compromising precise localization and boundary definition.

**Figure 4:**
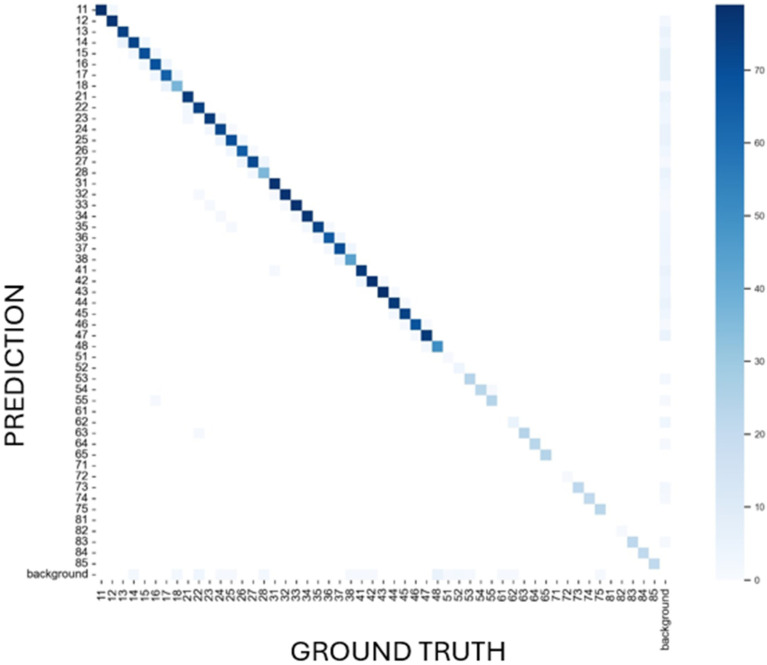
Normalised confusion matrix related to prediction of Ultralytics YOLO 11 (vertical axis) and ground truth (expert annotation, horizontal axis).

The normalized 52 × 52 confusion matrix (Figure 4) provided comprehensive visualization of model classification patterns, with ground truth annotations displayed on the x-axis and model predictions on the y-axis. Each cell represents the percentage of predictions for a given true versus predicted tooth class, with color intensity corresponding to classification accuracy. The matrix demonstrated predominantly diagonal activity with minimal off-diagonal misclassifications, indicating robust discriminative capability. The sparse off-diagonal elements and pronounced diagonal concentration confirmed effective model training with limited systematic errors.

## 4. Discussion

The present study demonstrates significant methodological and analytical advancements in AI-assisted tooth detection and segmentation specifically tailored for mixed dentition PRs. Our novel YOLOv11-based framework achieved robust performance while addressing critical gaps in the current literature through comprehensive dentition-type stratification and detailed error characterization. These findings support the rejection of our null hypothesis, confirming that AI-based DL models can achieve clinically acceptable accuracy levels for automated mixed dentition analysis.

AI in dentistry represents a paradigm shift from traditional diagnostic approaches to AI-augmented clinical decision-making. While conventional methods rely primarily on clinician expertise and subjective interpretation, AI enables data-driven analyses that enhance diagnostic accuracy and efficiency. This transition creates new opportunities, including earlier detection of developmental anomalies, reduction in inter-operator variability, and enhanced support for personalized treatment planning and monitoring of patient compliance [25]. However, it simultaneously raises ethical considerations related to data privacy, transparency of algorithmic processes, and the preservation of the dentist–patient relationship. Recent reports from the World Health Organization (2023) and the European Commission (2024) emphasize that AI in healthcare must be developed and implemented within a human-centered, transparent, and ethically robust framework [26,27]. Recommendations highlight the need for accountability mechanisms, continuous professional training, and regulatory oversight to ensure that AI systems enhance clinical decision-making without replacing the clinician–patient relationship [28].

Despite their potential, AI applications remain prone to errors. Misclassification, biased predictions, and opaque “black box” decision-making processes (i.e., situations in which an AI system generates outputs or recommendations without offering transparency regarding the internal reasoning or computational pathways that led to such results) can result in patient harm and loss of trust. The WHO (2023) recommendations suggest fostering “optimal trust,” where clinicians critically evaluate AI outputs rather than relying on them blindly [26,28]. Importantly, errors are not limited to algorithmic miscalculations but also derive from biased or incomplete training datasets, potentially exacerbating health inequalities. Moreover, echo chamber dynamics may amplify and reinforce a participant’s pre-existing beliefs through repetition and algorithmic biases. These risks highlight the need for careful design, monitoring, and human oversight in the deployment of conversational AI tools in clinical and health promotion contexts.

Future developments in AI should focus on explainability, interoperability, and integration into multidisciplinary care pathways. The WHO (2023) calls for investment in transparent models that provide not only predictions but also justifications for their recommendations [26]. Furthermore, the European Commission (2024) envisions AI as a catalyst for more resilient healthcare systems, particularly through predictive analytics, personalized medicine, and digital public health strategies [27].

Clinical adoption of AI remains hindered by challenges such as limited interpretability, data privacy concerns, regulatory fragmentation, and infrastructural disparities. Suggested solutions involve robust governance structures, mandatory reporting of serious incidents, transparent communication of risks and benefits to patients, and investment in equitable access to AI-enabled care [28]. Collaborative approaches, where clinicians, patients, policymakers, and technologists co-design AI solutions, are increasingly recommended as a strategy to align innovation with clinical and societal needs.

The systematic review by Maganur et al. (2024) synthesized evidence from 16 studies from 2018 to 2023 on AI models for tooth detection and numbering in dental radiographs [10]. AI models, predominantly based on convolutional neural networks, demonstrated very high performance, with some studies reporting precision up to 99.4% for detection and 98.0% for numbering. Nevertheless, heterogeneity in datasets and limited dataset size were recurrent limitations. Importantly, the review, serving as a lens through which to better analyze other types of studies [29], discusses the challenge of reliably recognizing deciduous teeth, citing morphological variability, overlapping structures, and limited training examples as major obstacles to robust performance in primary dentition. Overall, the review supports the potential of AI as a diagnostic adjunct while emphasizing the need for rigorous validation and enhanced methods specifically tailored to primary tooth recognition.

As can be seen in Table 3, the Ultralytics YOLOv11-based model in this study achieved competitive detection performance while also providing polygonal segmentation, thereby offering an analytical depth that extends beyond bounding-box detection. Beser et al. (2024), employing YOLOv5 on a dataset of 3854 pediatric PRs, reported slightly higher metrics for tooth detection and segmentation [19]. However, their work did not stratify performance by dentition type nor provide detailed error characterization, both of which are essential for clinical translation. Similarly, Kaya et al. (2022) obtained robust detection results with YOLOv4 on 4545 pediatric PRs, yet their methodology remained limited to bounding-box annotations without segmentation or differentiation between primary and permanent dentition [15]. Kilic et al. (2021), focusing exclusively on deciduous teeth with Faster R-CNN Inception v2, reported high accuracy, but their study lacked applicability to mixed dentition contexts [30]. More recently, Peker and Kurtoglu (2025) investigated YOLOv10 performance on a limited dataset (n = 200) and achieved satisfactory performance, yet again relying solely on bounding-box annotations [16].

Our stratified analysis highlighted a clear discrepancy between permanent and deciduous teeth, reflecting the inherent morphological variability, smaller size, and frequent overlap of primary teeth with developing permanent successors. This dentition-specific evaluation—largely absent in previous studies—provides clinically relevant insights by identifying priority areas for methodological refinement. Detailed error analysis further revealed that misclassifications were concentrated at dentition boundaries (notably teeth #51, #52, and #82), where overlapping anatomical structures reduce localization and boundary precision.

In the present study, the key methodological innovation was implementing a pre-annotation strategy utilizing transfer learning from an adult radiographic dataset. Initial training on 650 adult PRs from the publicly available DENTEX dataset enabled automatic generation of provisional labels for mixed dentition images, followed by systematic expert manual refinement. This hybrid two-stage approach represents a paradigm shift from conventional fully manual annotation workflows, significantly reducing labeling burden while maintaining high accuracy standards through targeted expert correction of deciduous teeth and developing permanent teeth that could be challenging to recognize. Unlike previous investigations that relied exclusively on manual annotation, our methodology optimizes annotation efficiency by exploiting the morphological similarities between fully developed permanent teeth across age groups, while ensuring accurate classification of primary dentition through specialized expert review. Moreover, the adoption of optimized hyperparameters specific to dental radiographic characteristics, such as limiting scale augmentation to preserve tooth proportions and disabling left-right flips to avoid numeration errors, further enhanced model performance.

This multifaceted approach represents a significant advancement toward developing robust AI systems optimized for pediatric dental imaging AI-based applications. Standardized automated analysis could reduce interpretation time and improve communication with patients through consistent diagnostic terminology, particularly relevant in healthcare systems with limited pediatric specialist access [31]. Accurate tooth identification represents an essential prerequisite for developing AI algorithms that analyze tooth position, orientation, and eruption trajectories. Looking ahead, in clinical practice, given that ectopic eruption poses significant orthodontic challenges when diagnosed late [32,33], AI-based predictive models could facilitate early detection when interceptive treatments remain most effective [9,32], rather than focusing on the detection of already impacted teeth [12,13]. Such predictive frameworks could substantially improve diagnostic efficiency while reducing clinician-dependent variability in mixed dentition evaluation.

Despite achieving competitive performance, some study limitations warrant acknowledgment. The dataset size could benefit from expansion to enhance generalizability across diverse pediatric populations, varied imaging protocols, and equipment specifications. The integration of multi-center validation studies would further strengthen the evidence base for clinical applications. Additionally, the performance gap between permanent and deciduous tooth detection suggests the need for specialized training strategies or augmented datasets focusing on primary dentition morphology. The model’s performance across different stages of dental development and varying numbers of present teeth (supernumerary teeth or agenesis) also remains to be fully characterized, representing an important area for future investigation that should include comprehensive evaluation across early mixed dentition, late mixed dentition, and transitional phases of dental development.

## 5. Conclusions

This study establishes the feasibility and clinical relevance of DL-based automated tooth recognition and segmentation in pediatric mixed dentition PRs. The developed system achieved excellent agreement with expert annotations, demonstrating a robust performance across diverse developmental stages, potentially improving diagnostic efficiency and reducing interpretive variability in mixed dentition PRs. The integration of an innovative hybrid pre-annotation strategy (leveraging transfer learning from a publicly available adult radiographic dataset, followed by systematic expert manual refinement) together with dentition-type performance stratification and detailed error characterization establishes a robust foundation for clinical translation while delineating specific areas for further advancement in AI-based applications for mixed dentition. Future multicenter validation studies will be essential for confirming the broader applicability and clinical utility of this approach across diverse healthcare settings and patient populations.

## Figures and Tables

**Table 1:** Bounding box performance (detection task). Precision, recall, mAP_0.5_, mAP50-95, and F1 scores by tooth number.

Class	Precision (P)	Recall (R)	mAP_0.5_	mAP50_95	F1 Score
11	0.989	0.993	0.995	0.708	0.991
12	0.962	0.980	0.990	0.627	0.971
13	0.983	0.987	0.992	0.645	0.985
14	0.961	0.974	0.985	0.520	0.967
15	0.988	1.000	0.995	0.573	0.994
16	0.991	0.926	0.960	0.611	0.957
17	0.973	0.919	0.941	0.669	0.945
18	0.946	0.990	0.980	0.620	0.968
21	0.982	0.987	0.994	0.669	0.984
22	0.986	0.986	0.987	0.598	0.986
23	0.991	0.987	0.987	0.626	0.989
24	0.963	1.000	0.995	0.489	0.981
25	0.958	0.987	0.993	0.622	0.972
26	0.976	0.993	0.995	0.718	0.984
27	0.987	0.908	0.920	0.632	0.946
28	0.991	0.975	0.993	0.624	0.983
31	0.976	0.993	0.995	0.491	0.984
32	0.979	0.993	0.995	0.509	0.986
33	0.990	0.993	0.995	0.554	0.992
34	0.976	0.980	0.993	0.550	0.978
35	1.000	0.982	0.995	0.550	0.991
36	0.993	0.974	0.988	0.694	0.984
37	0.976	0.981	0.994	0.664	0.978
38	0.957	0.986	0.988	0.555	0.972
41	0.987	0.991	0.995	0.474	0.989
42	0.982	0.974	0.985	0.502	0.978
43	0.988	0.993	0.995	0.575	0.991
44	0.964	0.974	0.984	0.557	0.969
45	0.977	0.993	0.993	0.493	0.985
46	0.958	0.986	0.987	0.617	0.972
47	0.919	0.942	0.980	0.683	0.930
48	0.967	0.985	0.992	0.623	0.976
51	0.553	0.800	0.635	0.179	0.654
52	0.643	0.778	0.780	0.400	0.704
53	0.948	0.921	0.981	0.490	0.934
54	0.914	0.950	0.965	0.464	0.932
55	0.963	0.941	0.972	0.457	0.952
61	0.846	1.000	0.995	0.455	0.917
62	0.874	0.693	0.849	0.319	0.773
63	0.963	0.877	0.962	0.470	0.918
64	0.955	0.917	0.940	0.484	0.936
65	0.931	1.000	0.994	0.563	0.964
72	1.000	0.634	0.863	0.349	0.776
73	1.000	0.947	0.965	0.410	0.973
74	0.959	0.950	0.980	0.387	0.955
75	0.930	0.866	0.963	0.401	0.897
82	0.809	0.714	0.793	0.310	0.759
83	0.874	1.000	0.994	0.448	0.933
84	0.954	0.975	0.992	0.339	0.965
85	0.956	0.969	0.990	0.372	0.962

**Table 2:** Segmentation performance. Precision, recall, mAP_0.5_, mAP50-95, and F1 scores by tooth number.

Class	Precision (P)	Recall (R)	mAP_0.5_	mAP50_95	F1 Score
11	0.975	0.980	0.974	0.586	0.978
12	0.955	0.973	0.977	0.415	0.964
13	0.976	0.980	0.983	0.420	0.978
14	0.922	0.935	0.933	0.351	0.928
15	0.907	0.918	0.895	0.259	0.913
16	0.978	0.914	0.932	0.492	0.945
17	0.940	0.888	0.896	0.495	0.913
18	0.865	0.905	0.891	0.399	0.884
21	0.963	0.966	0.974	0.368	0.965
22	0.946	0.945	0.943	0.386	0.945
23	0.937	0.933	0.930	0.352	0.935
24	0.865	0.899	0.853	0.276	0.882
25	0.952	0.980	0.984	0.468	0.966
26	0.963	0.980	0.987	0.563	0.971
27	0.974	0.896	0.910	0.481	0.933
28	0.917	0.902	0.921	0.407	0.909
31	0.884	0.900	0.836	0.225	0.892
32	0.925	0.939	0.915	0.294	0.932
33	0.983	0.987	0.986	0.414	0.985
34	0.916	0.920	0.889	0.297	0.918
35	0.986	0.968	0.975	0.415	0.977
36	0.987	0.968	0.986	0.537	0.977
37	0.950	0.955	0.956	0.534	0.953
38	0.948	0.976	0.977	0.370	0.962
41	0.947	0.951	0.944	0.257	0.949
42	0.889	0.881	0.844	0.234	0.885
43	0.975	0.980	0.979	0.407	0.977
44	0.938	0.947	0.939	0.370	0.943
45	0.957	0.972	0.968	0.308	0.965
46	0.953	0.980	0.981	0.383	0.966
47	0.900	0.923	0.952	0.490	0.911
48	0.932	0.949	0.951	0.414	0.940
51	0.555	0.800	0.635	0.162	0.655
52	0.644	0.778	0.780	0.230	0.704
53	0.933	0.905	0.968	0.316	0.919
54	0.842	0.875	0.837	0.203	0.858
55	0.945	0.923	0.945	0.338	0.934
61	0.678	0.800	0.642	0.193	0.734
62	0.632	0.500	0.554	0.140	0.558
63	0.838	0.763	0.738	0.149	0.799
64	0.887	0.852	0.853	0.339	0.869
65	0.916	0.983	0.981	0.465	0.948
72	0.771	0.490	0.616	0.204	0.599
73	0.824	0.781	0.687	0.187	0.802
74	0.934	0.925	0.960	0.307	0.930
75	0.930	0.866	0.965	0.310	0.897
82	0.655	0.571	0.612	0.095	0.610
83	0.851	0.973	0.959	0.232	0.908
84	0.783	0.800	0.748	0.127	0.792
85	0.913	0.924	0.949	0.258	0.918

**Table 3:** Comparison of recent studies on automated analysis of pediatric panoramic radiographs and the present work. The table summarizes dataset size, dentition stage, task (detection, segmentation, enumeration), model type and main reported metrics.

Study	n (PRs)	Age/Dentition	Task(s)	Model	Detection mAP_0.5_/F1	Segmentation mAP_0.5_/F1
Kaya et al., 2022 [15]	4545	Pediatric, mixed	Detection + numbering	YOLOv4	0.92/0.91	-
Beser et al., 2024 [19]	3854	Pediatric, mixed	Det. + Segm.	YOLOv5	0.98/0.99	0.98
Peker et al., 2025 [16]	200	Pediatric, mixed	Detection	YOLOv10	0.968/0.919	-
Kilic et al., 2021 [30]	1125	Pediatric, deciduous	Detection + numbering	Faster R-CNN	0.93/0.91	-
Our work	250	Pediatric, mixed	Det. + Segm. + Enumeration	YOLOv11-seg	0.963/0.953	0.89

## Data Availability

The data presented in this study are available on request from the corresponding author due to privacy.

## Supplementary Materials

The following supporting information can be downloaded at: https://www.mdpi.com/article/10.3390/diagnostics15202615/s1, Table S1: STROBE Statement—Checklist of items that should be included in reports of cross-sectional studies.

## Abbreviations

The following abbreviations are used in this manuscript:

AI	Artificial Intelligence
PR	Panoramic Radiographs
DL	Deep Learning
LLM	Large Language Model
FDI	Federation Dentaire Internationale
DIBINEM	Department of Biomedical and Neuromotor Sciences
DISI	Department of Computer Science and Engineering
MICCAI	Medical Image Computing and Computer-Assisted Intervention
DENTEX	Dental Enumeration and Diagnosis on Panoramic-X-rays
mAP	mean Average Precision
IoU	Intersection over Union
